# Supplementary material for: Dust tides and rapid meridional motions in the Martian atmosphere during major dust storms
Source: Nat Commun. 2020 Jan 30;11:614. doi: 10.1038/s41467-020-14510-x (PMC6992627; doi:10.1038/s41467-020-14510-x)
Supplement: Supplementary file 1 — Supplementary Information [file 41467_2020_14510_MOESM1_ESM.pdf]

Supplementary Information for:

Dust Tides and Rapid Meridional Motions in the Martian Atmosphere During Major Dust Storms

by Wu et al.

Supplementary Figures

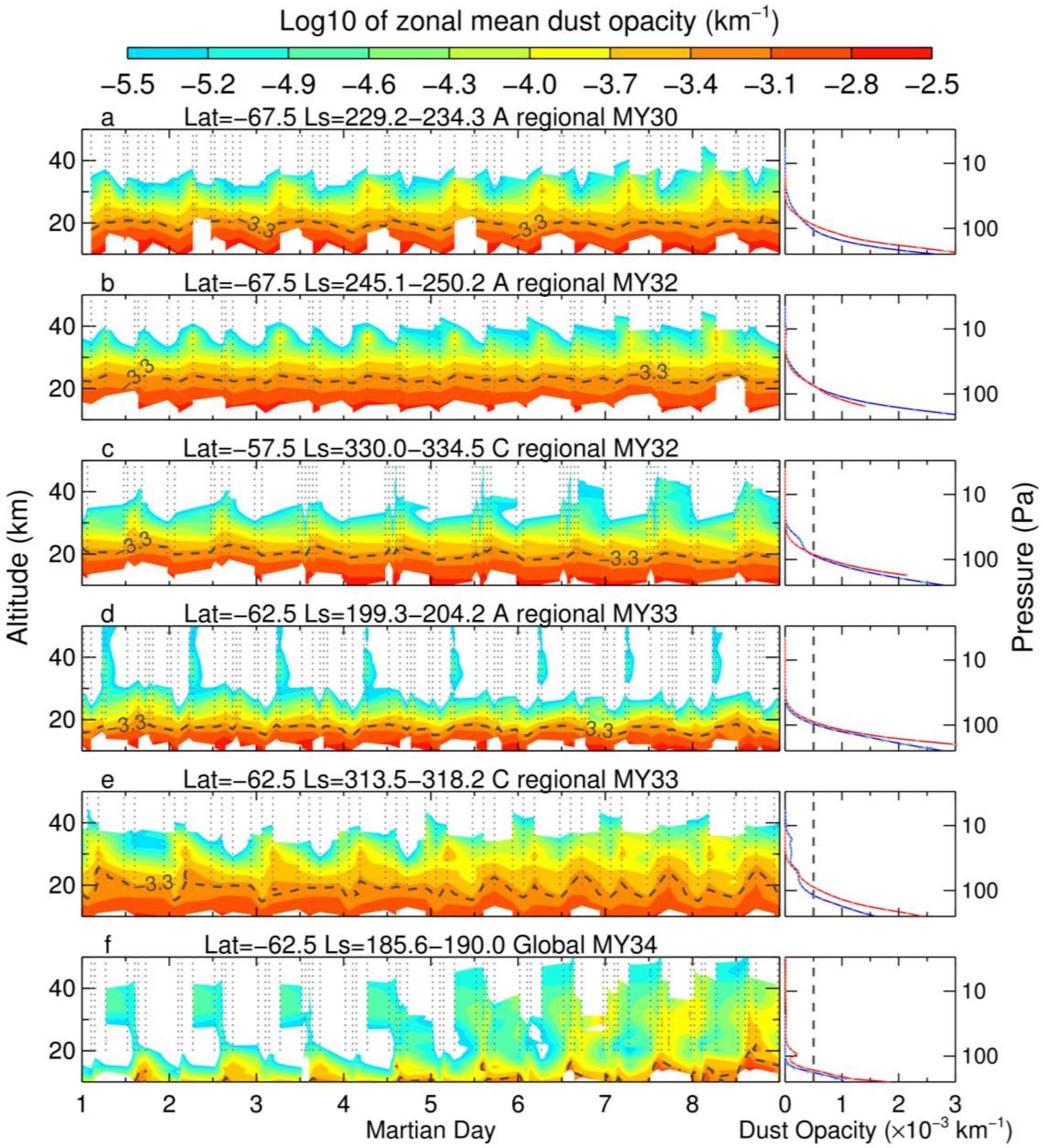

Supplementary Fig. 1 | Evolutions of the zonal-mean dust opacity before or after major dust storms.

Lack of strong diurnal variations in the zonal-mean dust opacity at 463 cm^{-1} (22 μm) before or after major dust storms shown in Fig. 1. a, c, d, e for prior to regional dust storms, b for after regional dust storms and f for prior to global dust storm. Plotting scheme is same as Fig. 1.

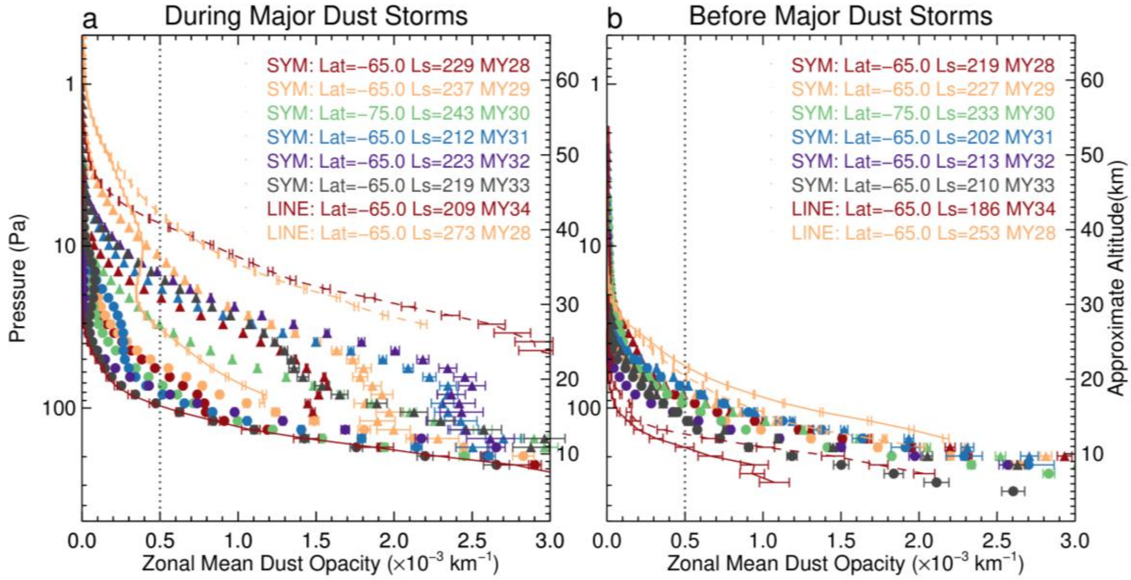

Supplementary Fig. 2 | Zonal-mean dust opacity profiles during and before major dust storms.

Zonal-mean dust opacity profiles during (a) and before (b) major dust storms in MY 28-34. The diurnal variations are shown by comparing the zonal-mean dust opacity in the daytime (~3 p.m., represented by triangles for regional dust storms and dash lines for global dust storms) and nighttime (~3 a.m., represented by circles for regional dust storms and solid lines for global dust storms). The retrieval uncertainties are given by the horizontal error bars. The vertical dot lines correspond to the dust opacity of 0.0005 determining the dust height.

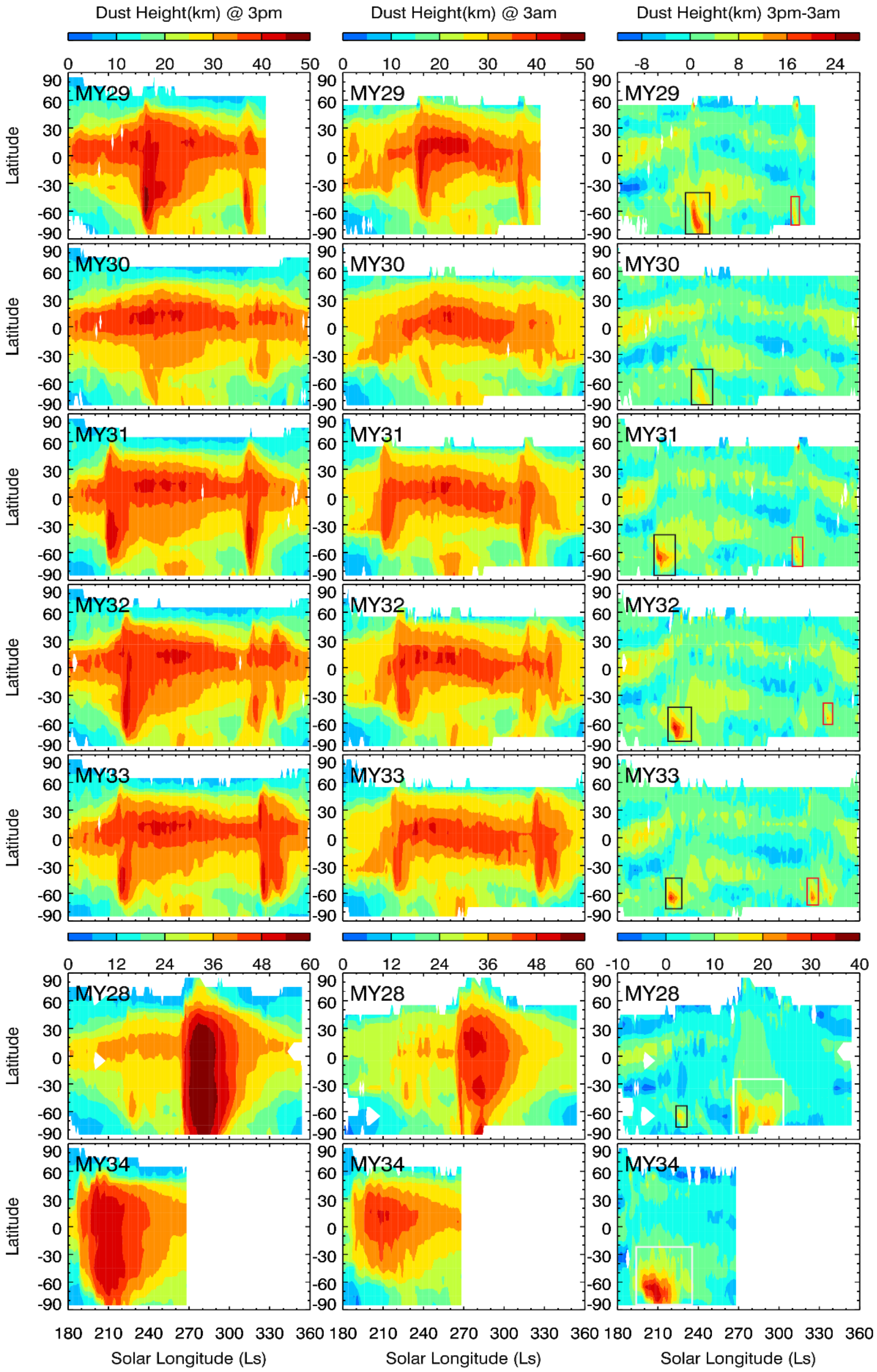

Supplementary Fig. 3 | The diurnal variability in dust height during major dust storms.

The structures of the dust height in the daytime (left column) and the nighttime (middle column) and the day-night difference (right column) in the storm seasons for 7 MYs (L_s=180°-360°). The A-season and C-season regional storms and global dust storms are highlighted with the black, red and white boxes respectively. The A-season regional storm is stronger than the C-season regional storm but much weaker than the global dust storm.

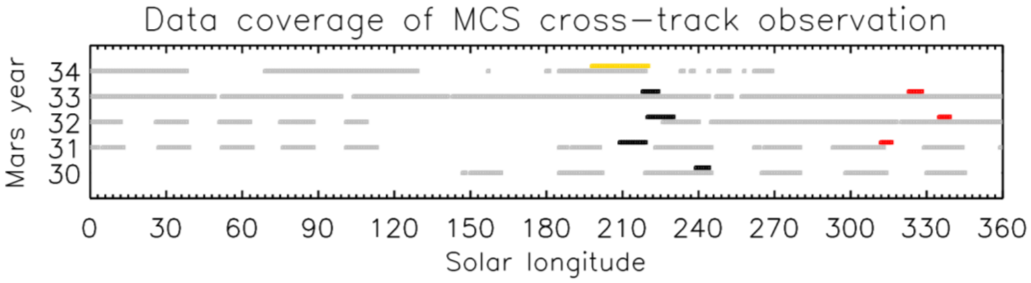

Supplementary Fig. 4 | Data coverage of MCS cross-track observation. Available periods (grey) with the cross-track observational strategy of MCS during MYs 30-34. The periods of A-season and C-season regional and global dust storms are indicated by black, red and gold colors respectively.

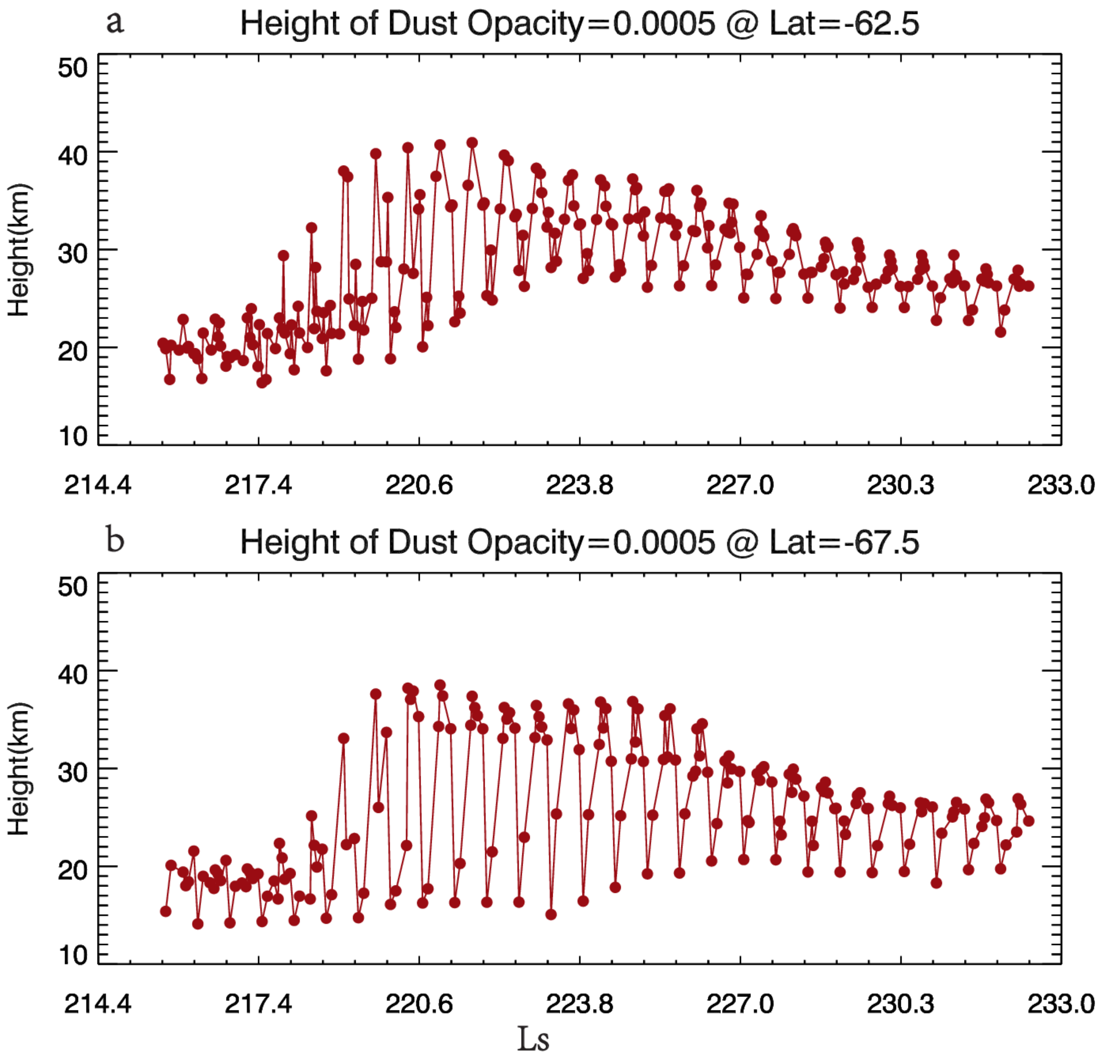

Supplementary Fig. 5 | Dust height evolution with time resolution in hours.
a, at 62.5°S and b, at 67.5°S during A-season regional dust storm

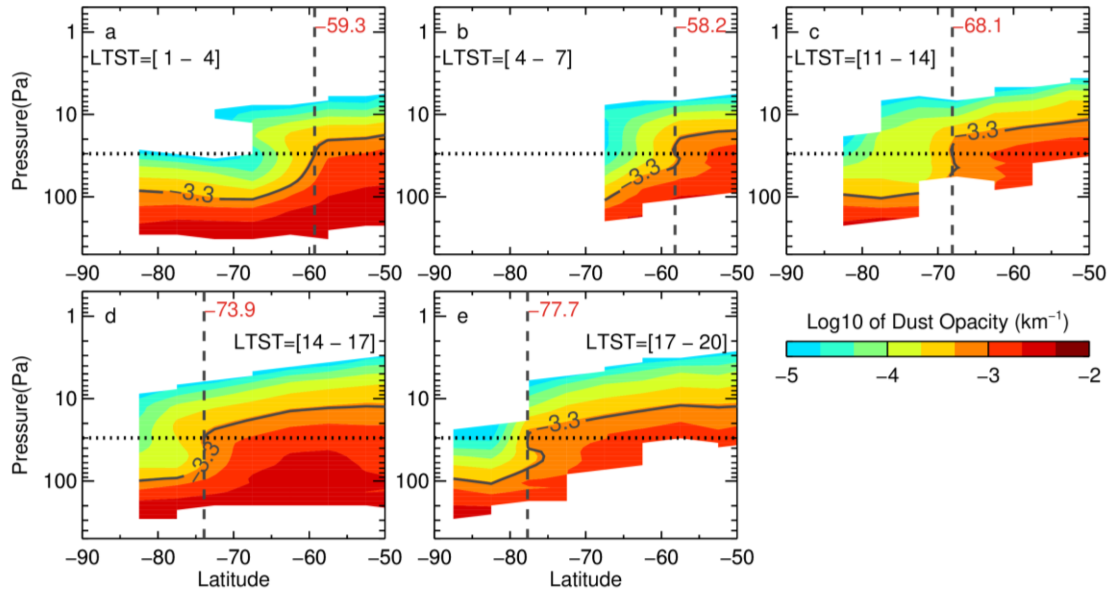

Supplementary Fig. 6 | The diurnal meridional motion of the dust front.
The vertical and meridional structures of high latitude summer hemisphere dust opacity during the peak time (L_S=220°-221.9°) of A-season regional dust storm in MY 33. The local time values are labeled in the figures. The black solid contour lines indicate the dust height with the opacity of 0.0005. The horizontal dot lines at 25 Pa are used to determine the latitude of the dust front. We averaged the data for 4 sols and then binned with a time window of 3 hours to increase the latitudinal and longitudinal coverages. A more detailed description on the data binning strategy is shown in Supplementary Fig. 7. For zonal average procedure, a bottom line of no less than 2/3 of the total longitude bins (8 of 12) is ensured, except for data at 67.5°S in 11:00-14:00 LTST (**c**) which only has coverage of 1/2 of the total longitude bins.

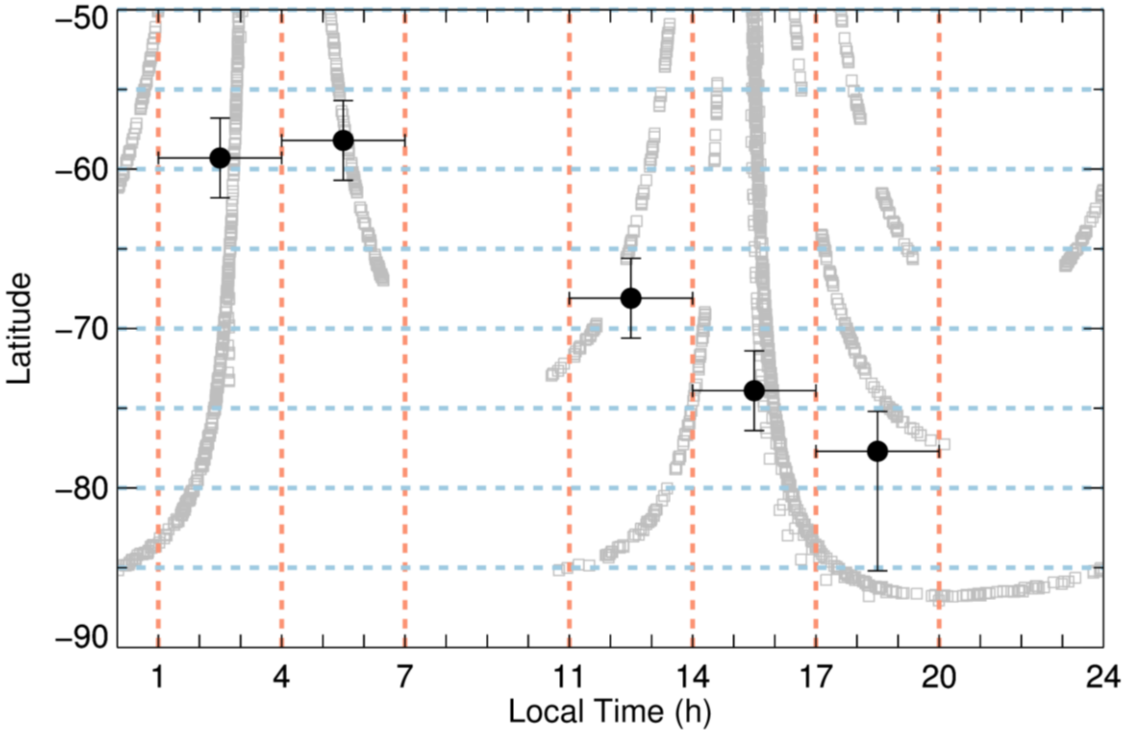

Supplementary Fig. 7 | The data binning strategy for Supplementary Fig. 6.
Grey boxes indicate the local time coverage of MCS measurements versus latitude for the cross-track observation sequence during the period (L_s=220°-221.9°) used for Supplementary Fig. 6. The blue horizontal dash lines depict the latitude bin boundaries. The red vertical dash lines depict the local time bin boundaries for 1, 4, 7, 11, 14, 17, 20 LT corresponding to Supplementary Fig. 6. All dust opacity data within a certain bin grid (circled by two adjacent red lines and two adjacent blue lines) are averaged as the value of the latitude and local time corresponding to the center of the bin grid. The 5 black dots represent the latitude values of the dust front at corresponding local times determined in Supplementary Fig. 6. The potential biases caused by the uneven distributed data point within each bin grid is represented by the horizontal (equal to the local time bin interval) and vertical (according to the latitude bin interval) error bars for reference.

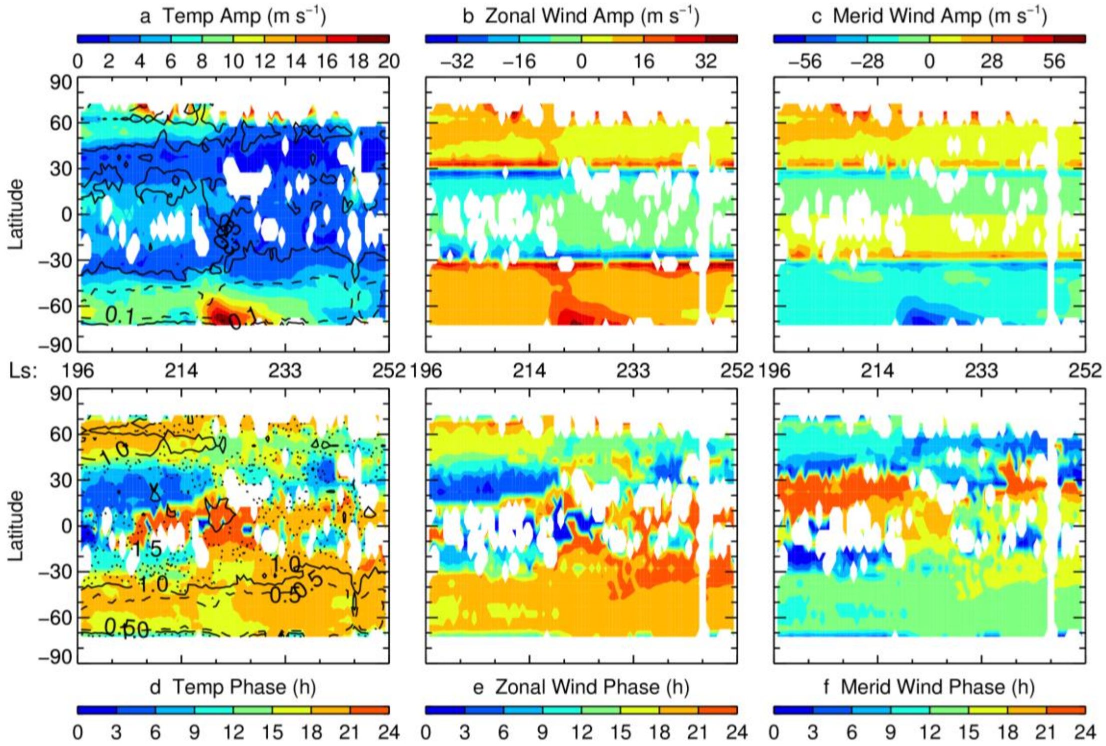

Supplementary Fig. 8 | The evolution of temperature, zonal and meridional wind in migrating diurnal tide.

The DW1 amplitudes (a, b, c, in units of K for a and m/s for b, c) and phases (d, e, f in unit of hour) of temperature (a, d), zonal wind (b, e) and meridional wind (c, f) during A-season regional dust storm in MY33. The period runs from Ls= ~196° to ~252° (90 sols, 10 sols for each tick interval of x-axis). The error contours in a represent the ratio of 1-sigma uncertainty to amplitude while in d represent the 1-sigma uncertainty in unit of hour. Since the winds DW1 are computed from idealized polarization relations and it is unrealistic to estimate the uncertainties introduced by the assumptions used in the polarization relations, all errors of the winds DW1 are not presented.

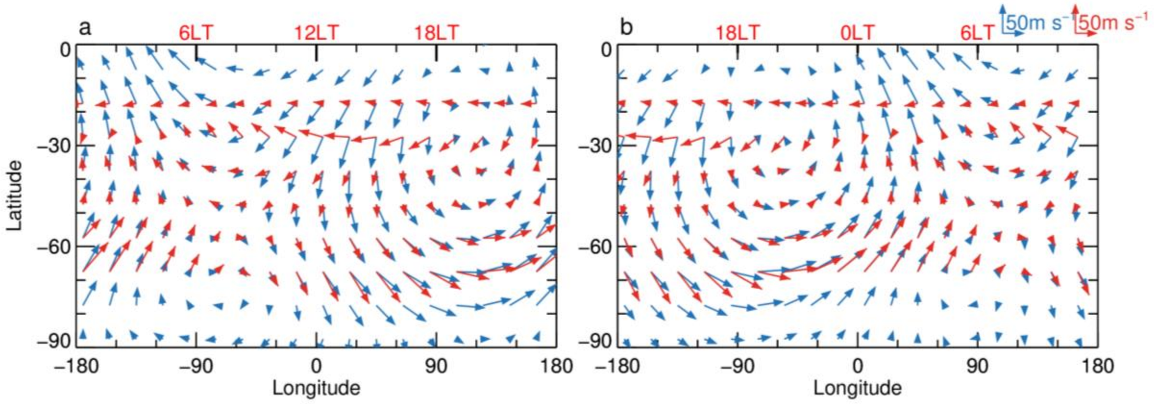

Supplementary Fig. 9 | The derived and simulated diurnal-varying horizontal wind distributions.

Diurnal variation of the horizontal wind fields at 50 Pa at a, 12:00 UT (universal time) b, 00:00 UT. Red vectors for the derived winds from classical gradient and tidal wind theories during A-season regional dust storm (L_s=220°-221.9°) in MY 33. Blue vectors for the simulated winds from MCD 5.3 during A-season regional dust storm (L_s=225°) in MY 32. The MCD 5.3 provides no results for MY 33 so we use MY 32 instead. Note that A-season regional dust storms in MY 32 and MY 33 have similar occurring time (Supplementary Fig. 3).

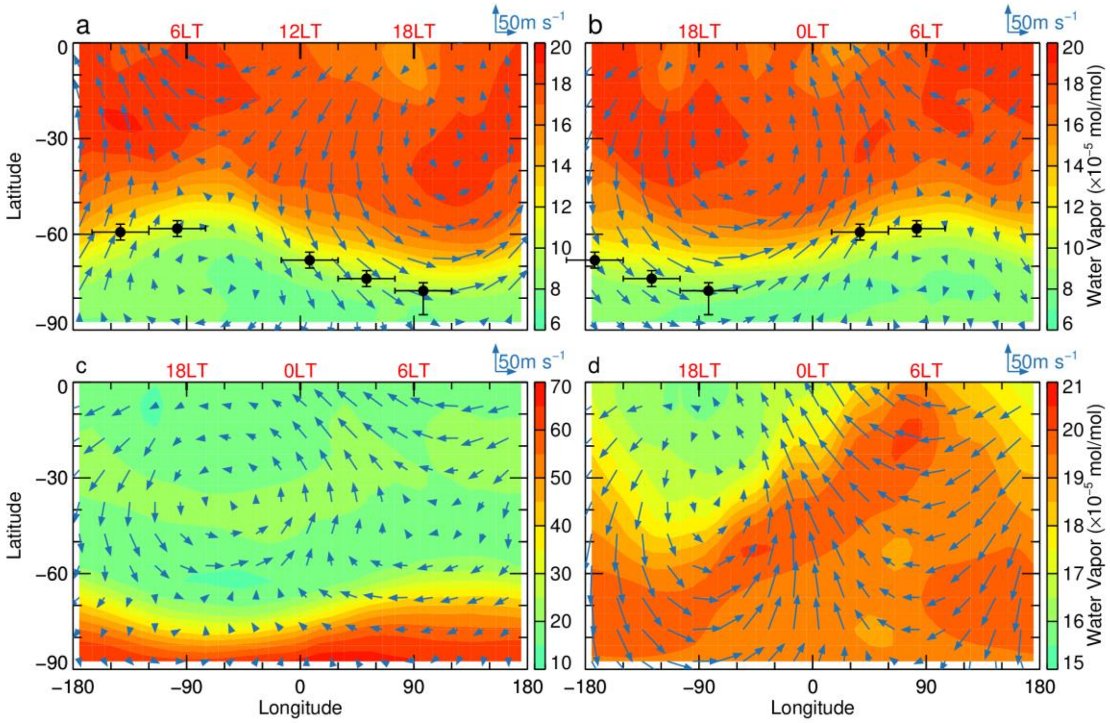

Supplementary Fig. 10 | The simulated diurnal-varying horizontal wind and water vapor distributions.

Diurnal variation of the horizontal wind fields (blue vectors) and water vapor distribution (colors) at 50 Pa from MCD 5.3. a, 12:00 UT, L_s=225° in MY32. b, 00:00 UT, L_s=225° in MY32. c, 00:00 UT, L_s=280° in MY32. d, 00:00 UT, L_s=280° in MY28. The black dots with error bars in a and b show the latitude values of the observational dust front (see Fig. 4 and Supplementary Fig. 6). Note that the wind vector pattern and magnitude in a and b are comparable to Fig.4 c and d as suggested in Supplementary Fig. 9. The good agreement between the wave pattern of the water vapor front (yellow belts in a and b) and the observational dust front may just be a coincidence because the global distribution of water vapor has clearly seasonal trend as introduced in the Discussion section. This good match cannot be treated as a solid correlation between dust and water vapor. We also note here that the color bar scales are different for different plots.
